# Supplementary material for: TRIM5α restricts poxviruses and is antagonized by CypA and the viral protein C6
Source: Nature. 2023 Aug 9;620(7975):873–80. doi: 10.1038/s41586-023-06401-0 (PMC10447239; doi:10.1038/s41586-023-06401-0)

nature portfolio

https://doi.org/10.1038/s41586-023-06401-0

Supplementary information

TRIM5α restricts poxviruses and is antagonized by CypA and the viral protein C6

In the format provided by the authors and unedited

Fig. 1

1b)

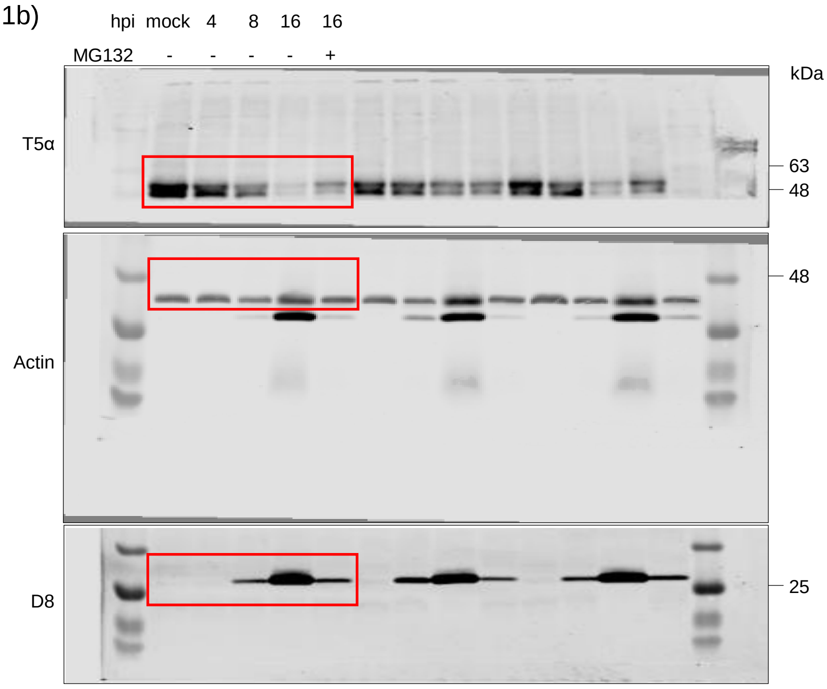

1c)

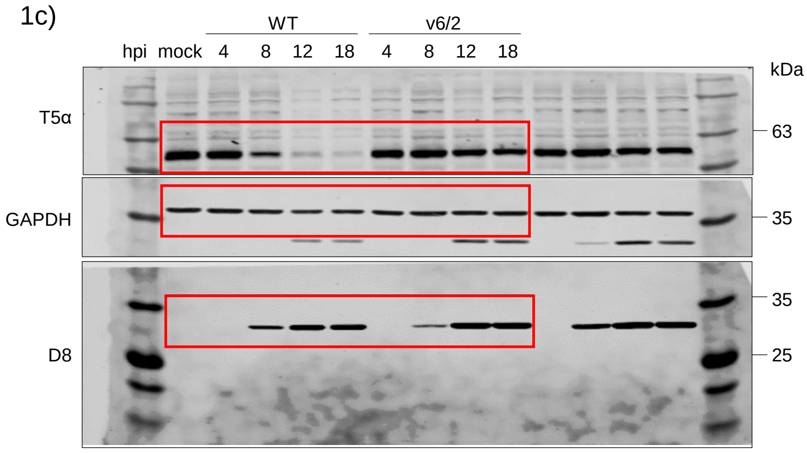

1d)

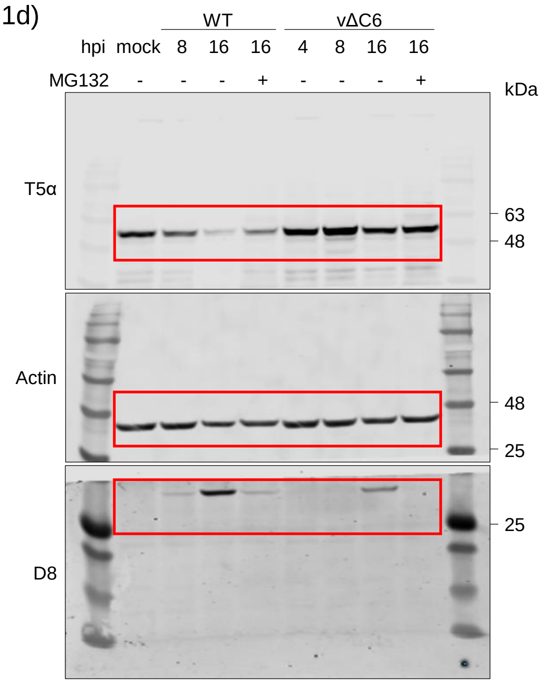

1e)

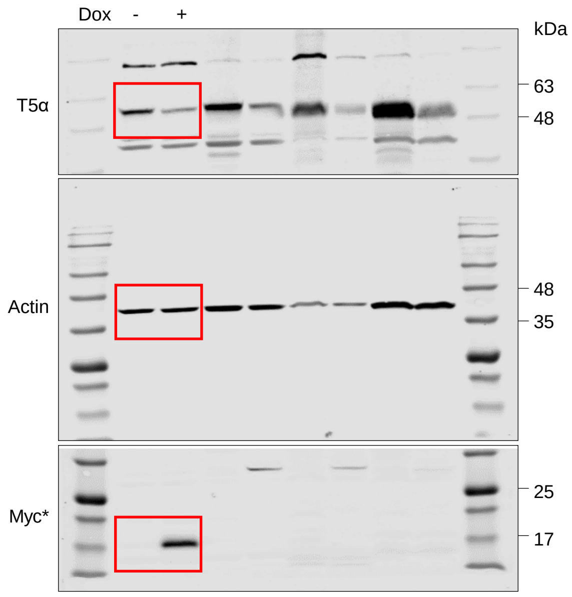

Fig. 1

*These proteins were run on separate gels

1f)

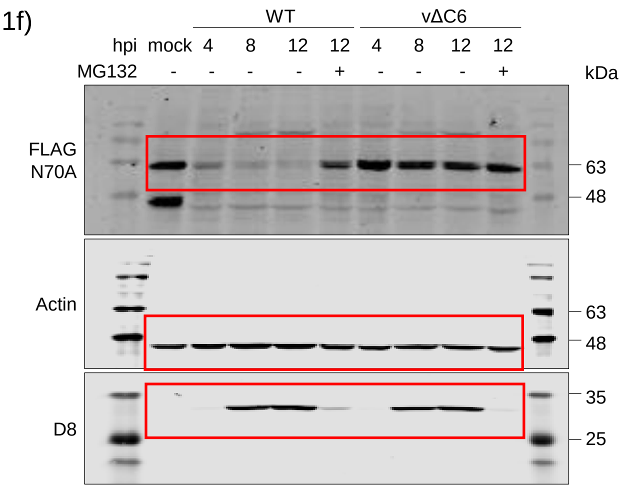

1g)

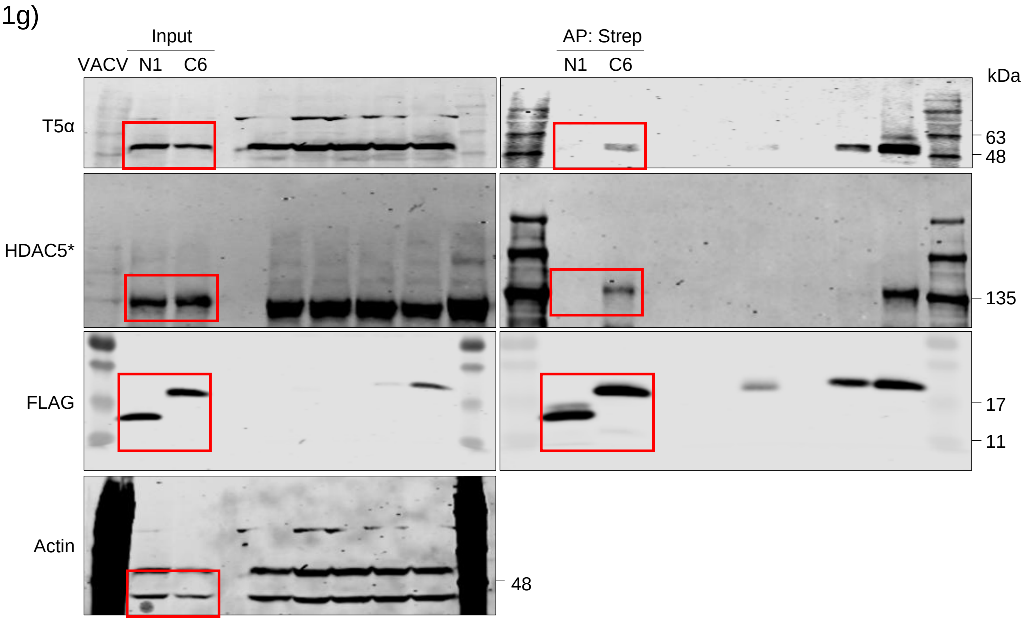

1h)

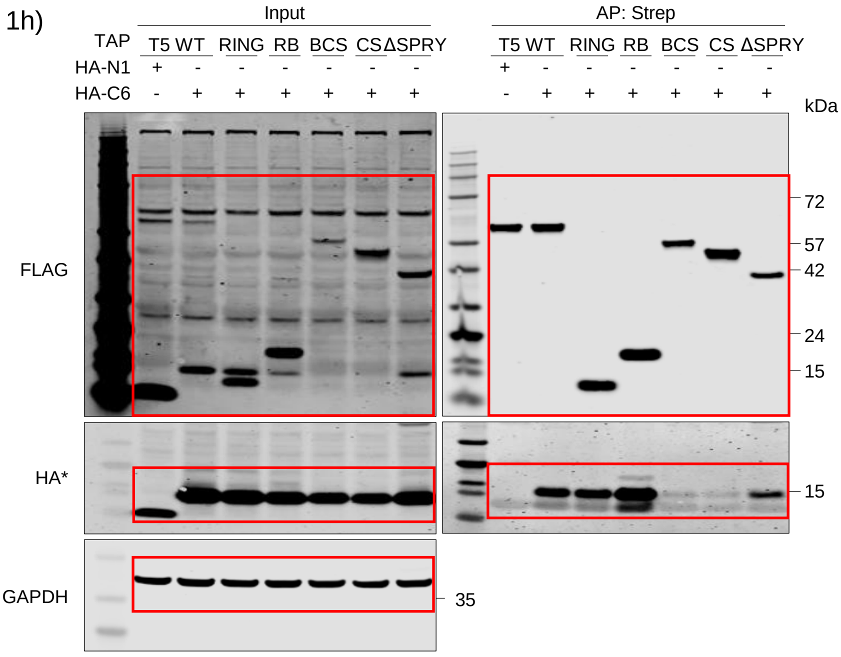

Fig. 4

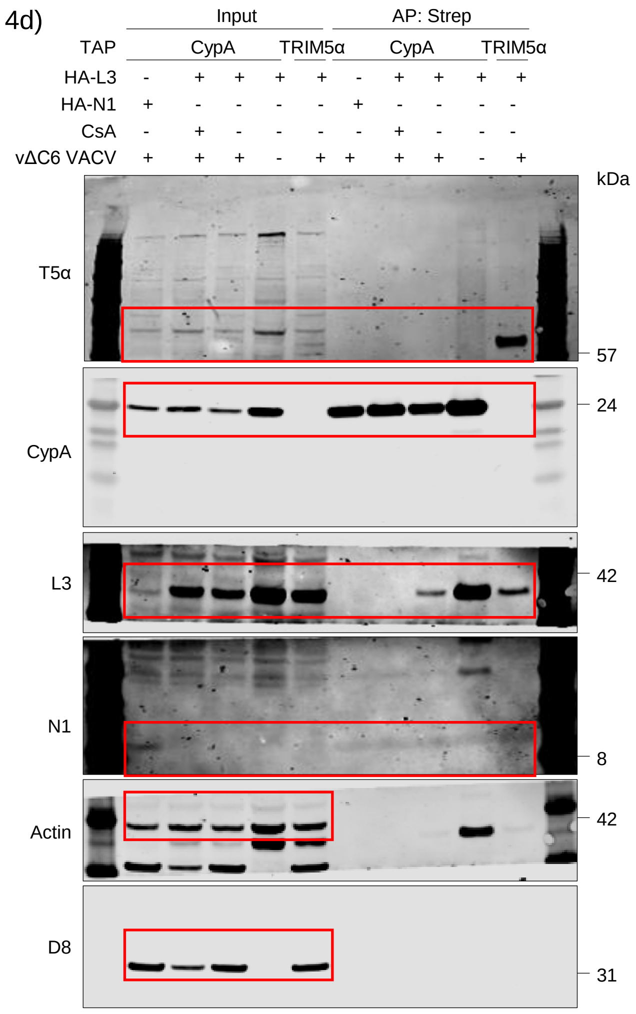
4d)
Input
AP: Strep
TAP
CypA
TRIM5α
CypA
TRIM5α
HA-L3
HA-N1
CsA
vΔC6 VACV
T5α
CypA
L3
N1
Actin
D8
kDa
57
24
42
8
42
31

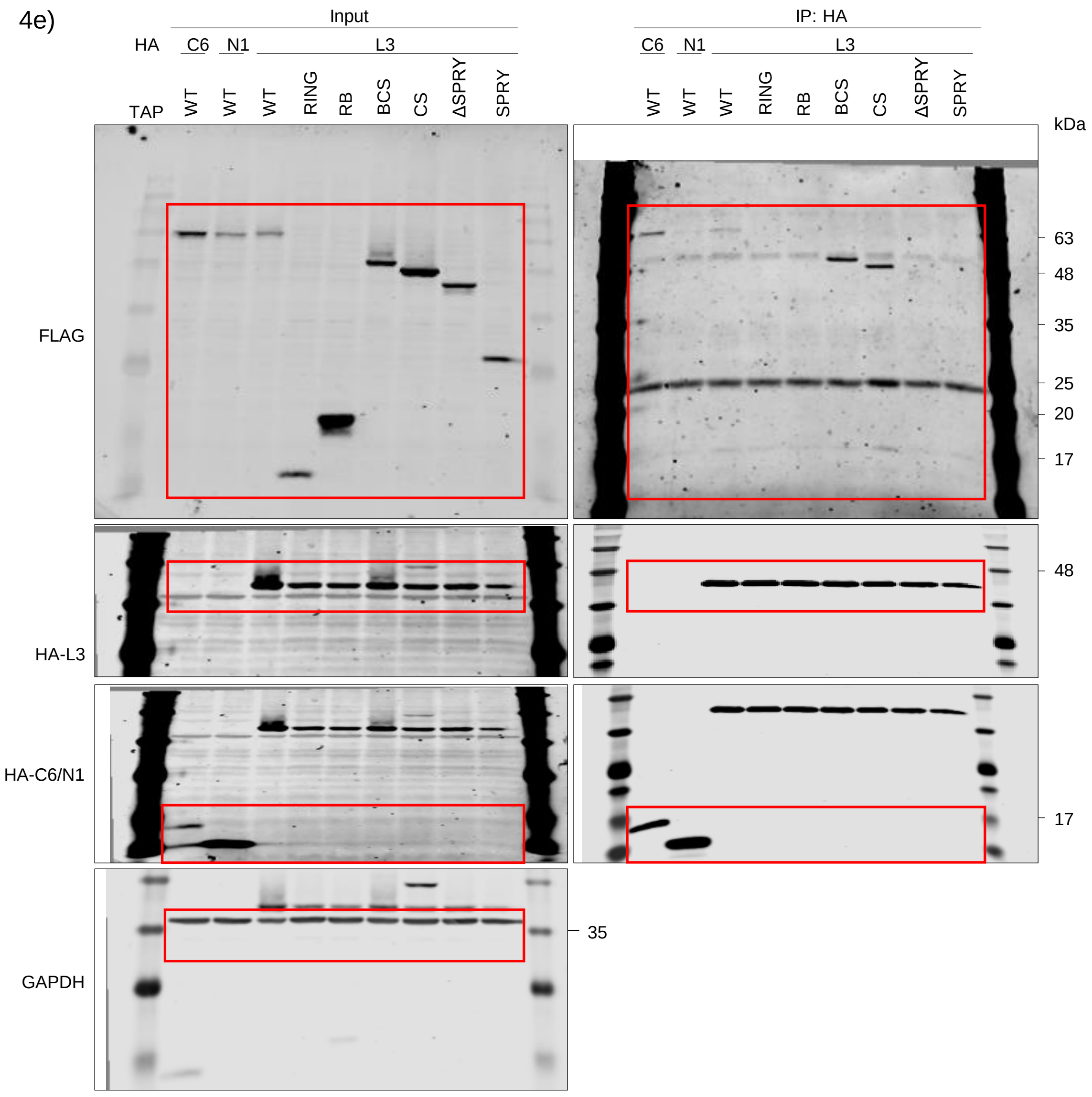
4e)
Input
IP: HA
HA
C6
N1
L3
TAP
WT
WT
WT
RING
RB
BCS
CS
ΔSPRY
SPRY
kDa
FLAG
HA-L3
HA-C6/N1
GAPDH
63
48
35
25
20
17
48
17
35

Fig. 4

4g)

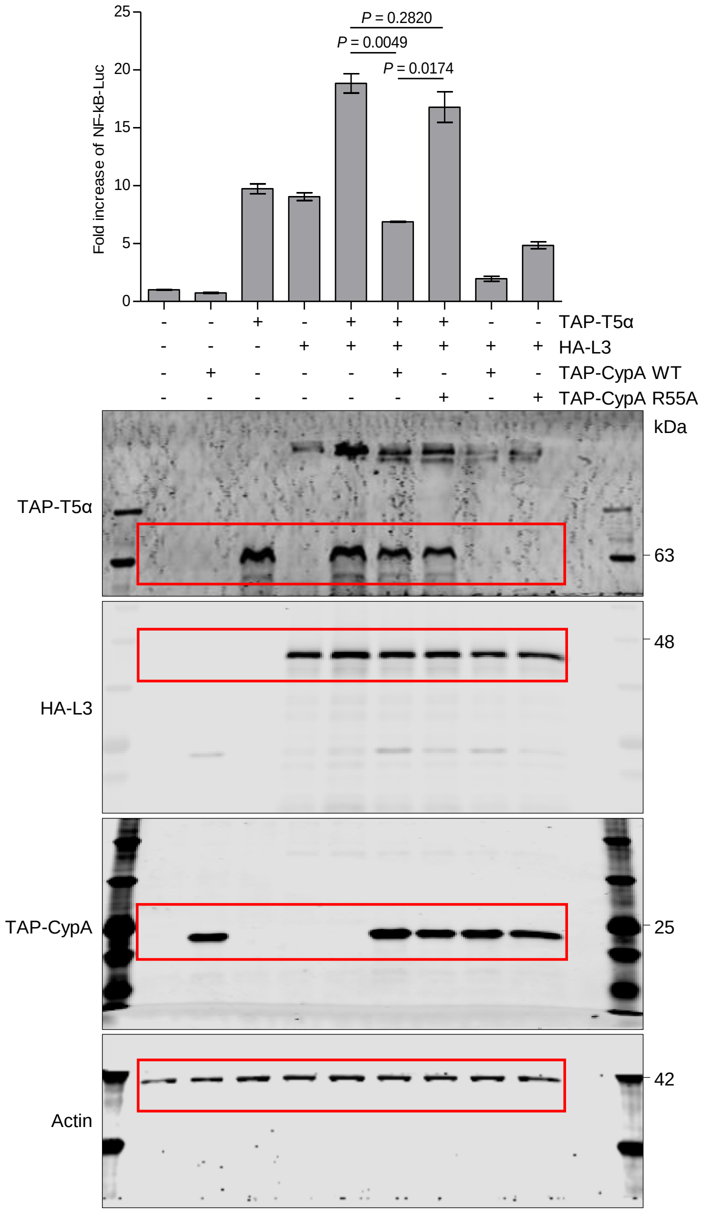

Fold increase of NF-kB-Luc
25
20
15
10
5
0
P = 0.2820
P = 0.0049
P = 0.0174
TAP-T5α
HA-L3
TAP-CypA WT
TAP-CypA R55A
kDa
TAP-T5α
63
48
HA-L3
TAP-CypA
25
42
Actin

Fig. 5 *These proteins were run on separate gels

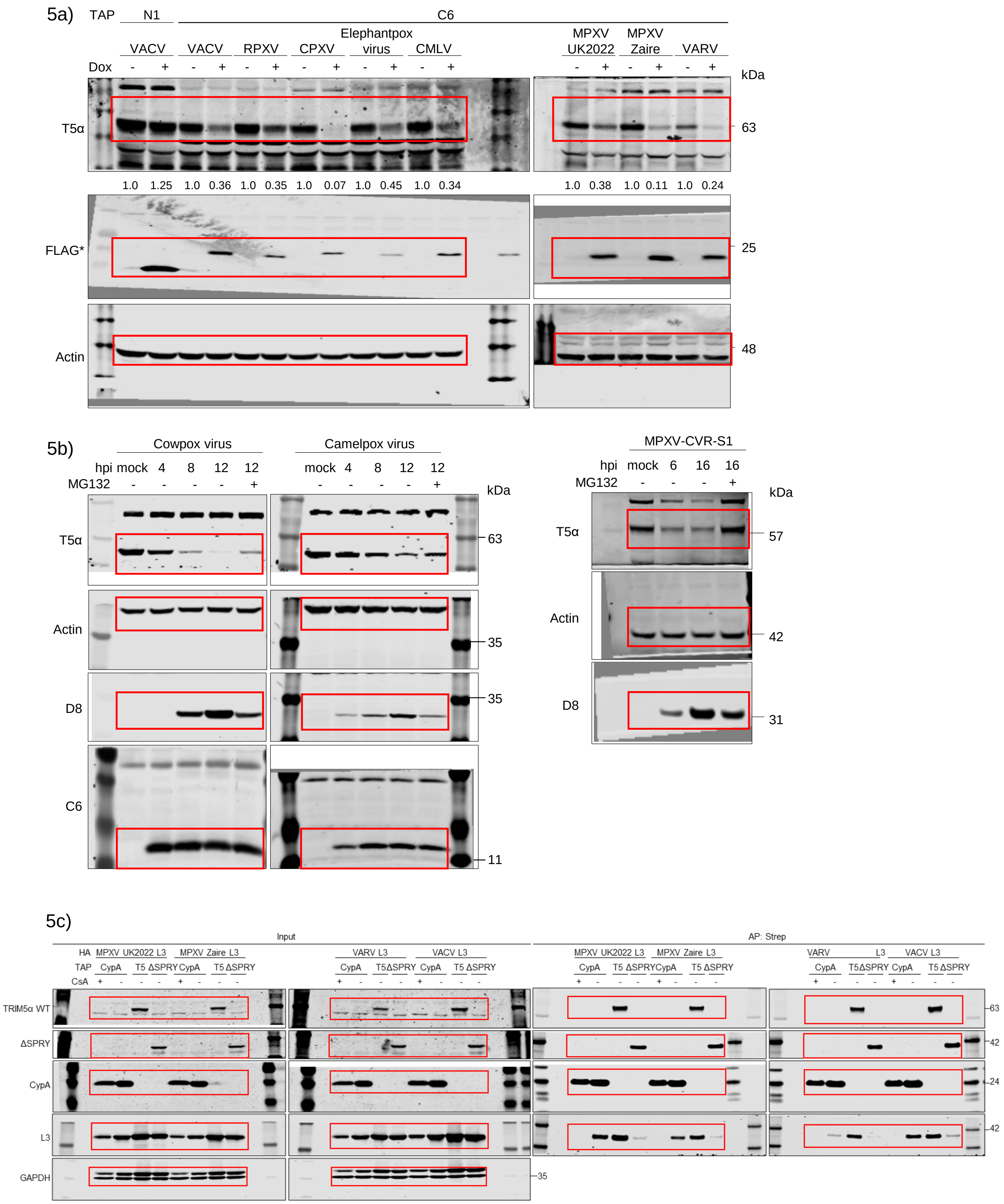

Extended Data Fig. 1

*These proteins were run on separate gels

ED1a)

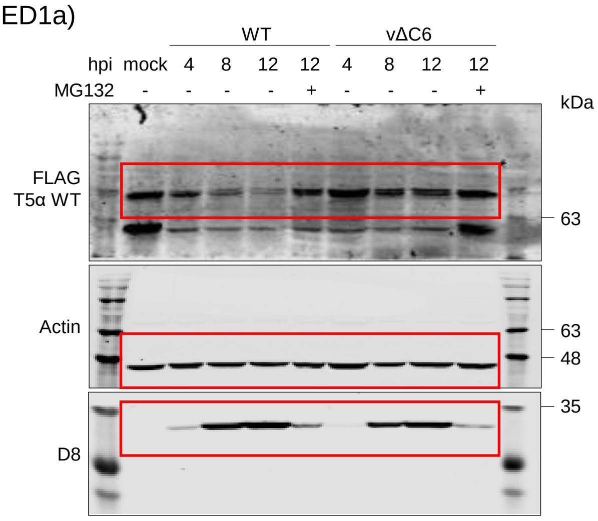

ED1b)

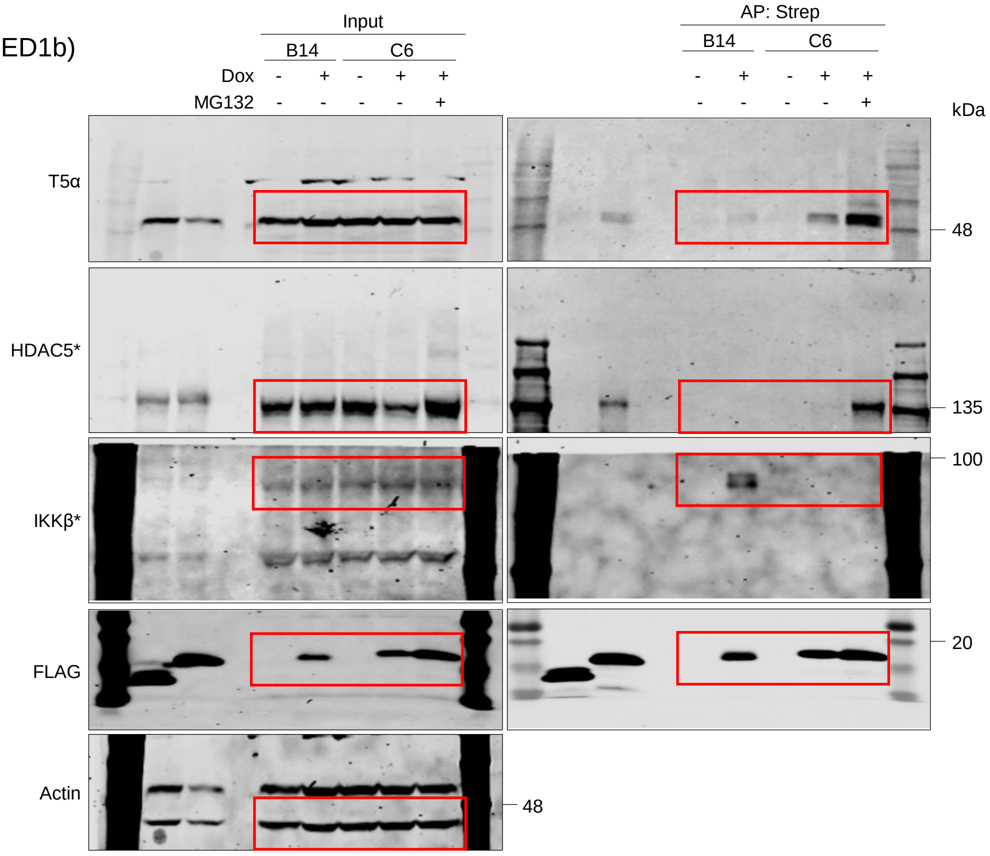

ED1c)

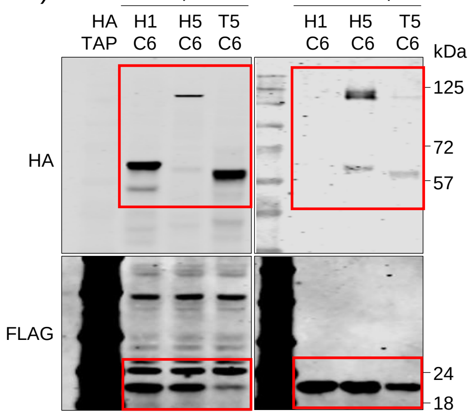

Extended Data Fig. 1

ED1d)

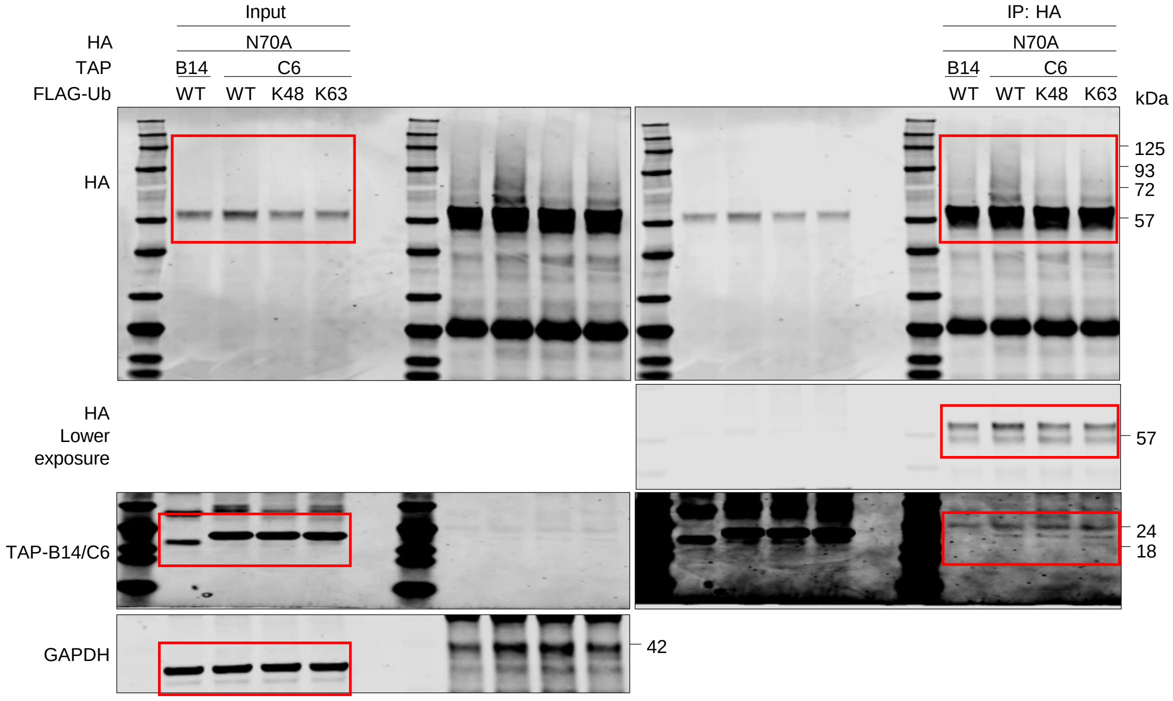

Extended Data Fig. 2

ED2a)

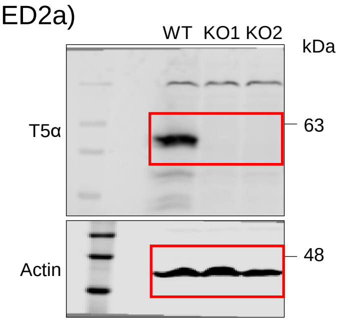

ED2b)

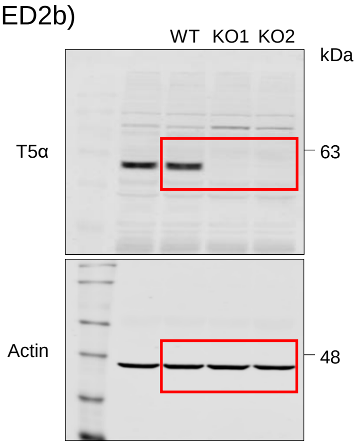

ED2g)

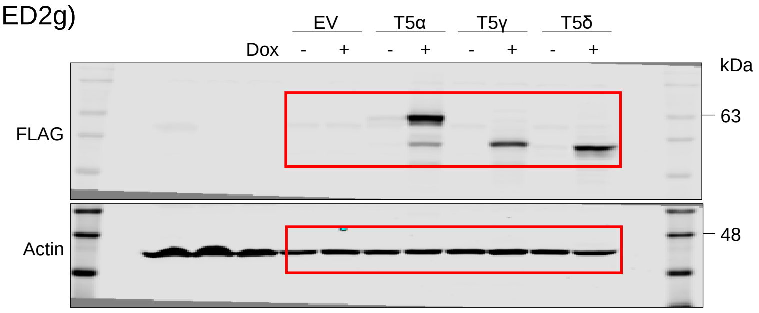

ED2l)

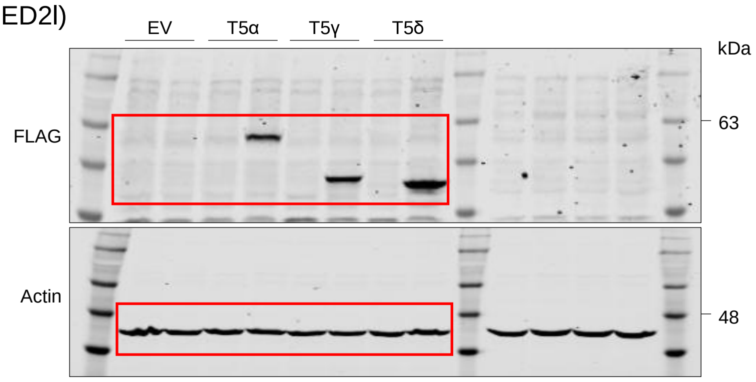

ED2o)

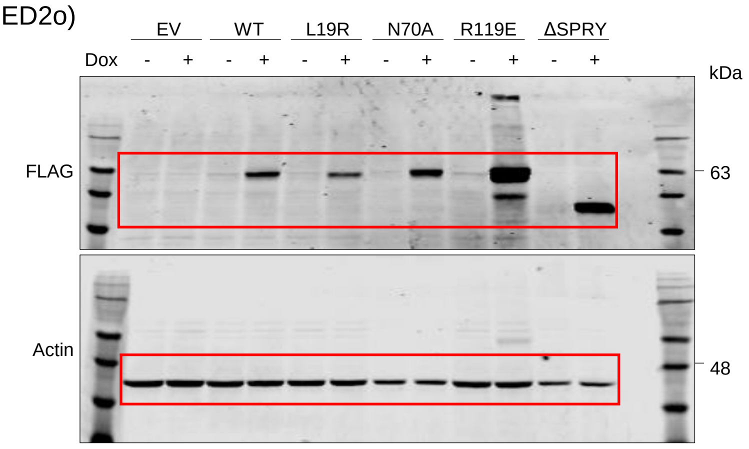

Extended Data Fig. 3

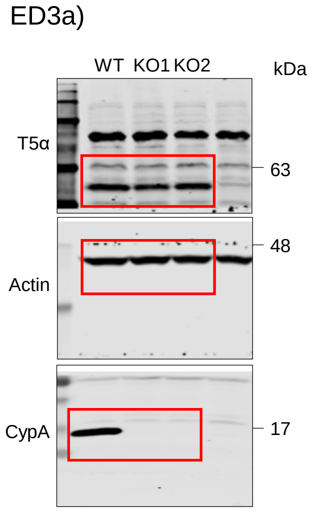

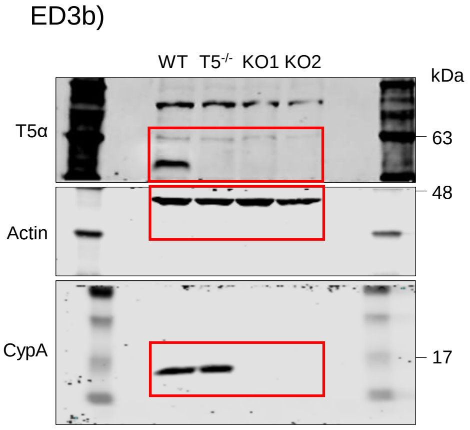

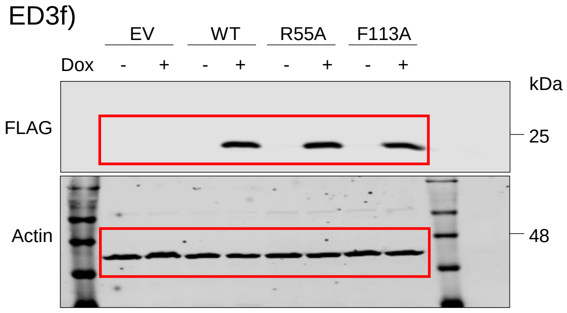

Extended Data Fig. 4

ED4a)

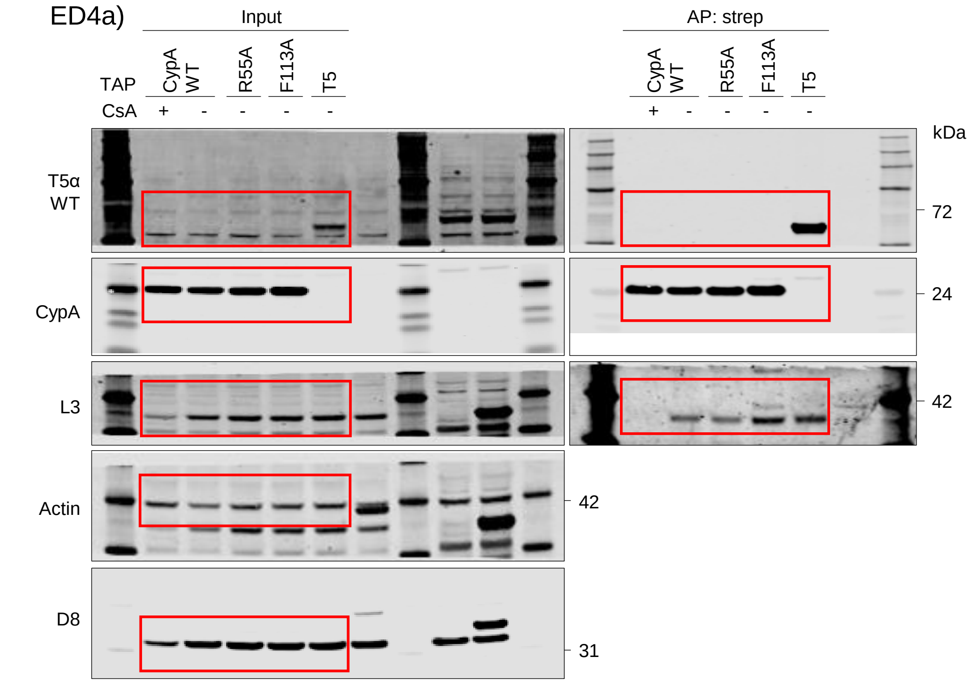

ED4b)

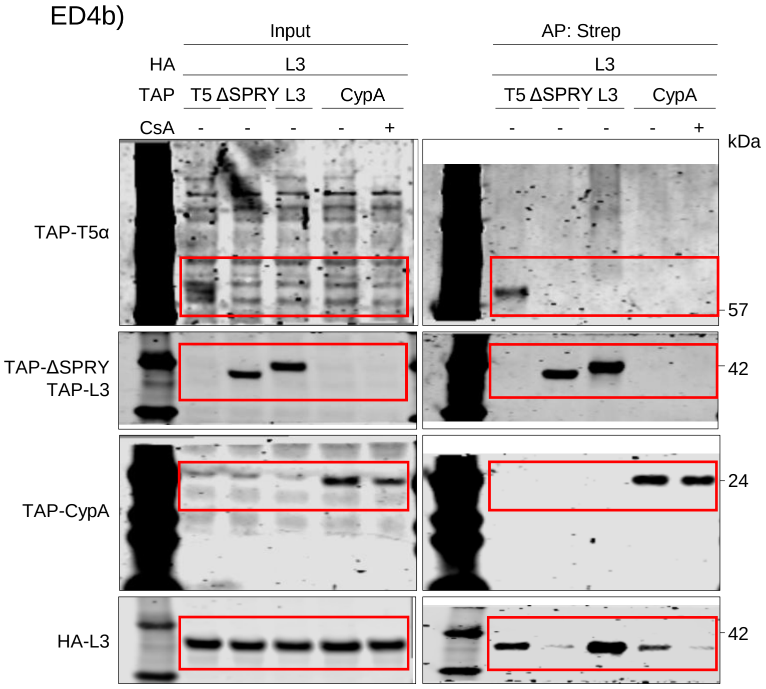

Extended Data Fig. 5

*These proteins were run on separate gels

ED5a)

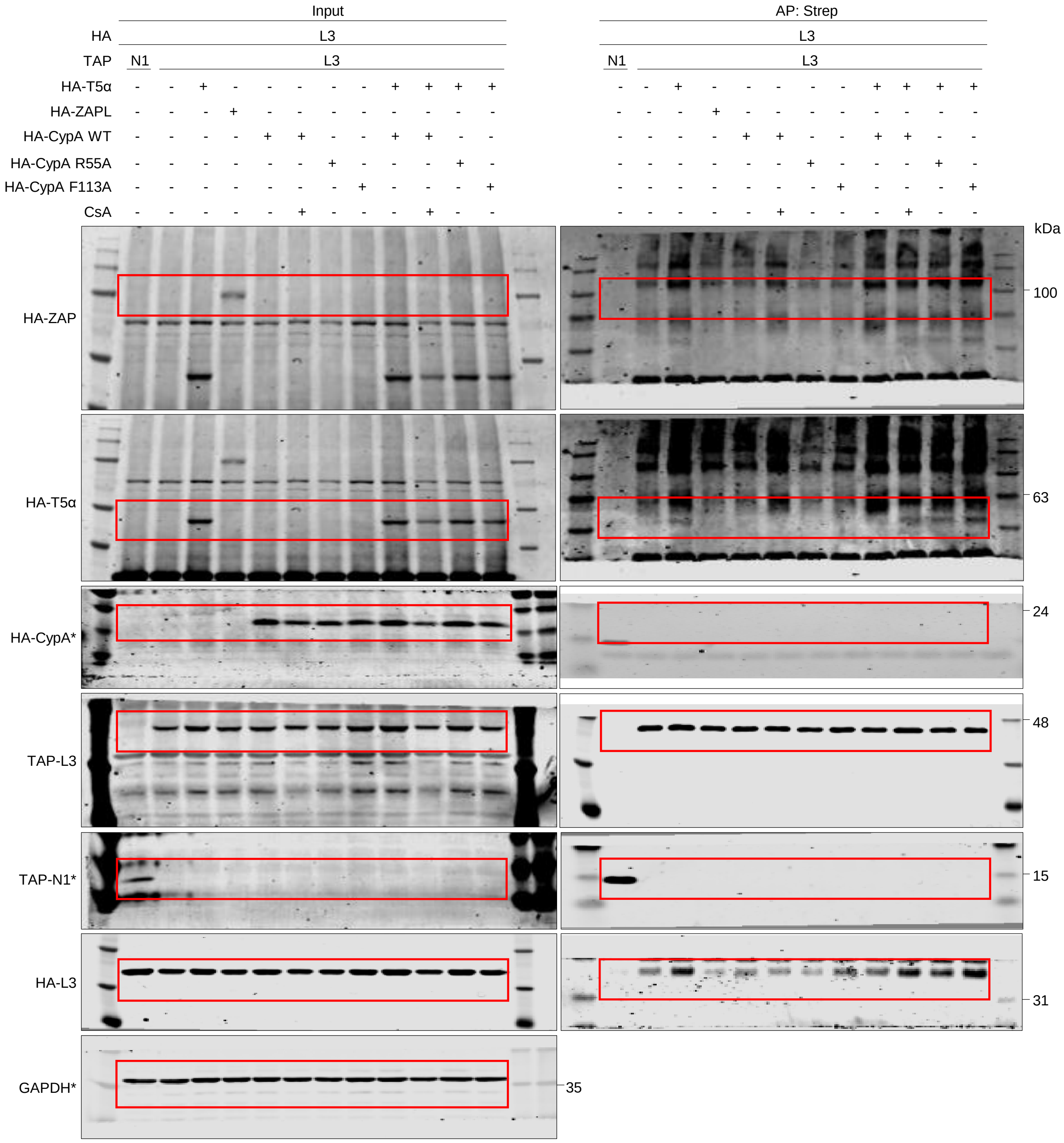

Extended Data Fig. 5

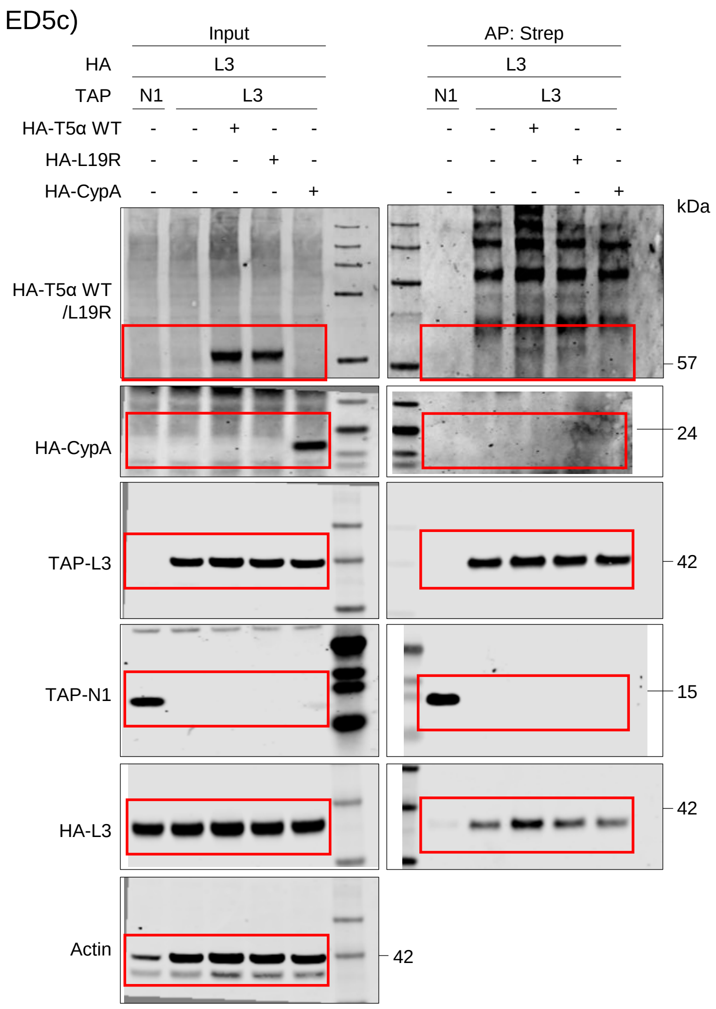

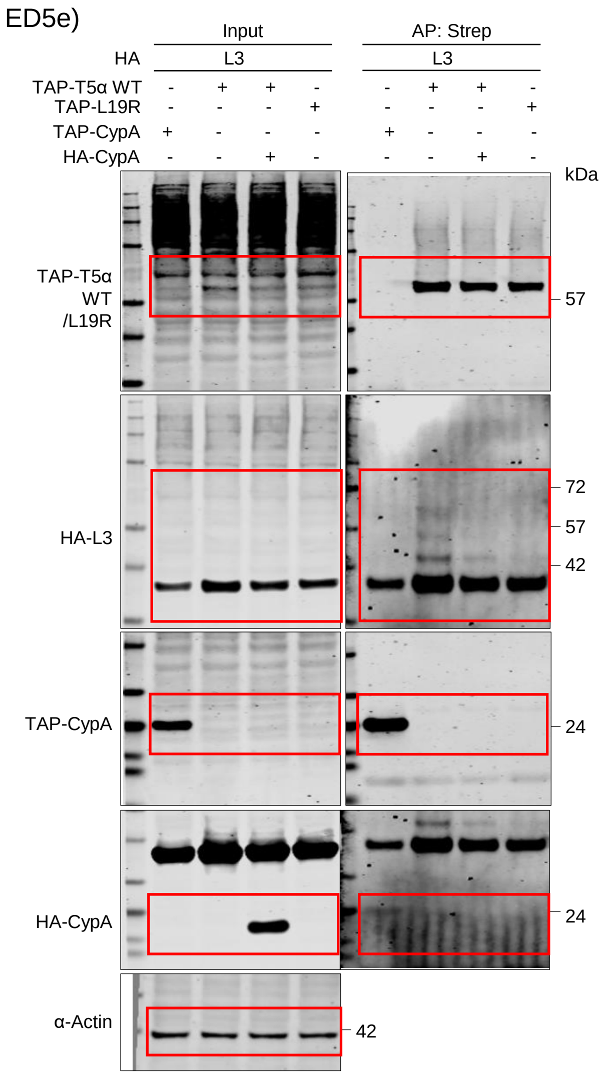

Extended Data Fig. 6

*These proteins were run on separate gels

ED6c)

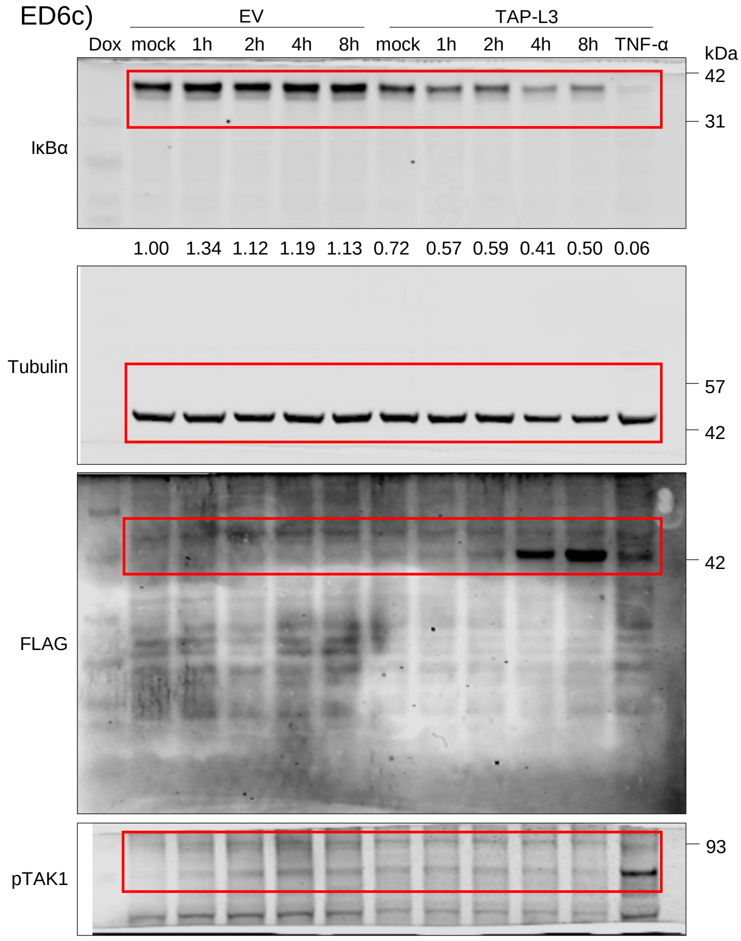

ED6e)

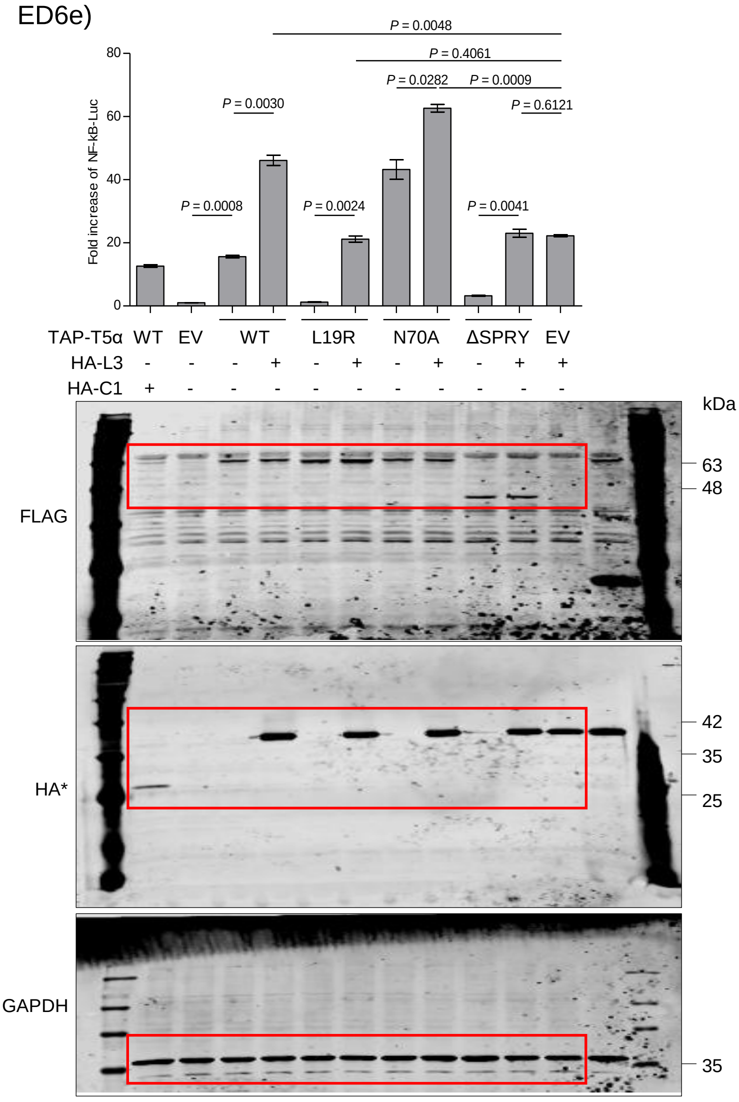

Extended Data Fig. 7

*These proteins were run on separate gels

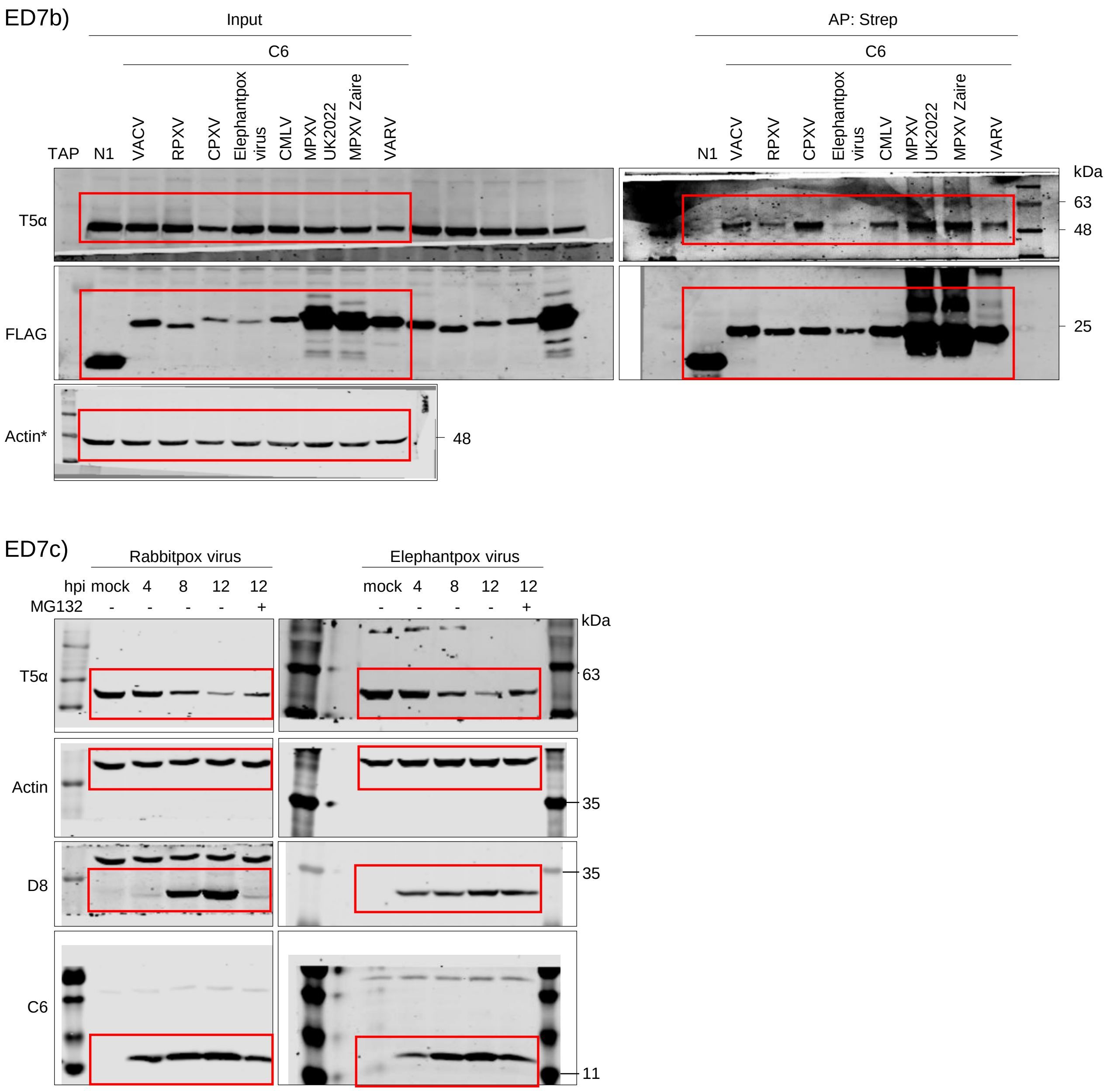

Extended Data Fig. 8

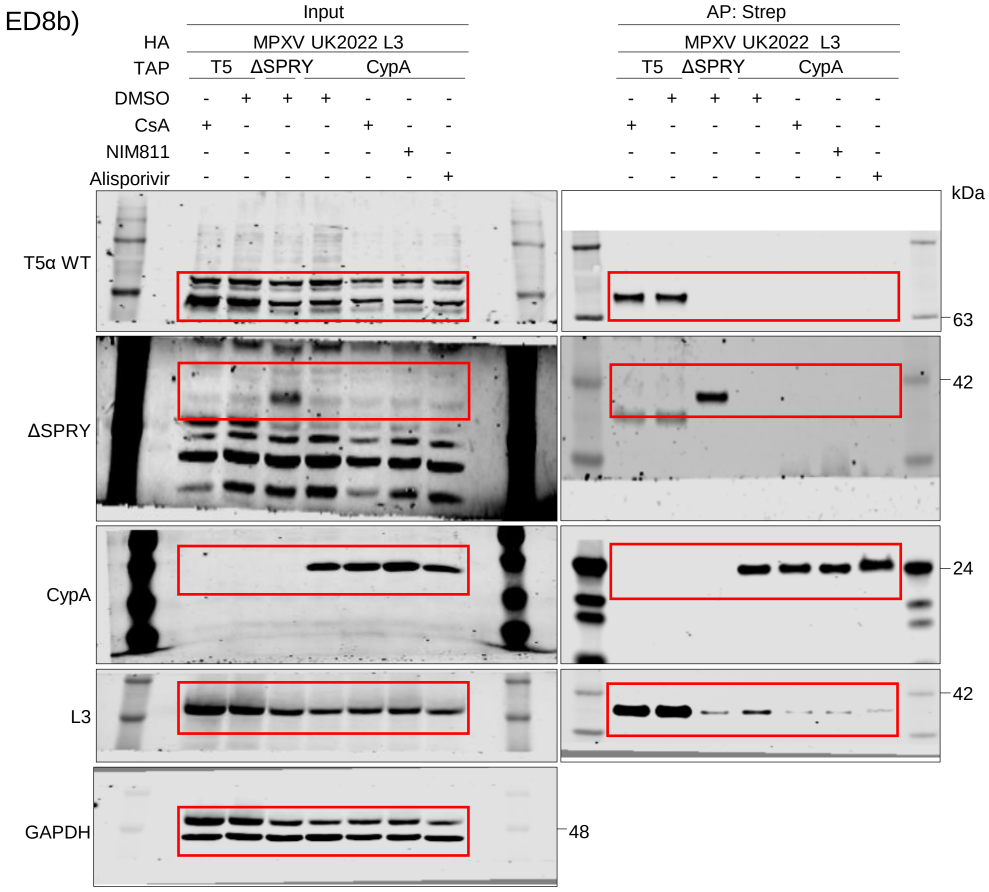

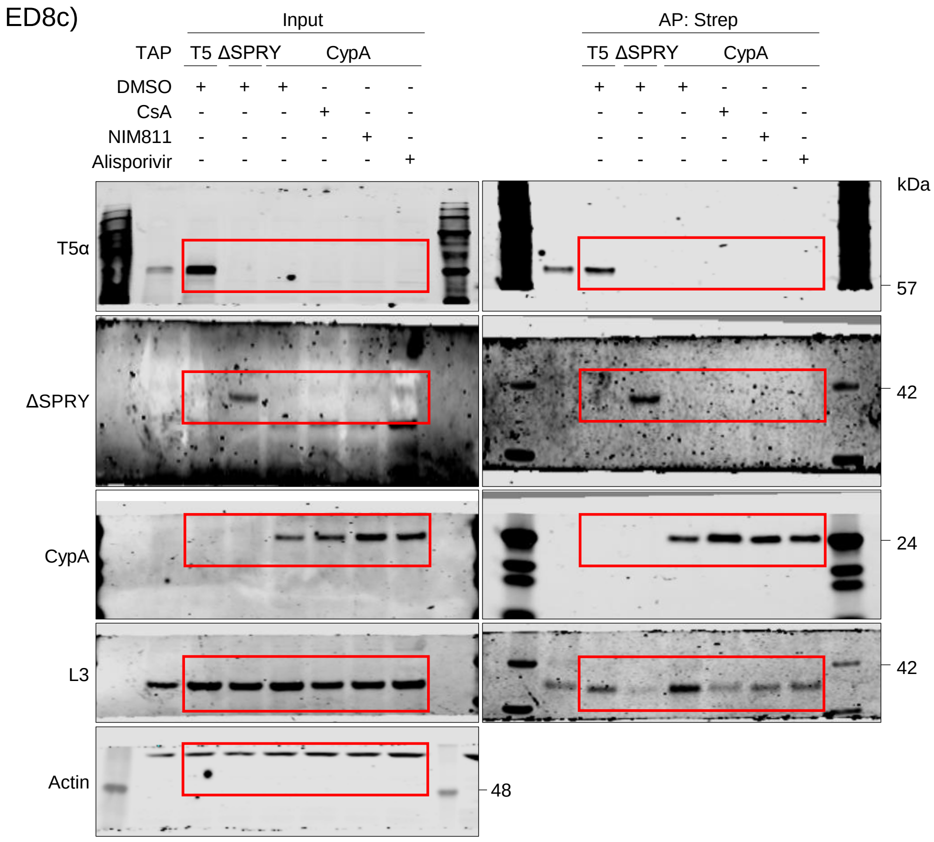

Supplement: Supplementary file 1 — Raw images of all immunoblots in the main and Extended Data Figures. These are presented in chronological order representing the order they appear in the figures. [file 41586_2023_6401_MOESM1_ESM.pdf]
