# Supplementary material for: TRIM5α restricts poxviruses and is antagonized by CypA and the viral protein C6
Source: Nature. 2023 Aug 9;620(7975):873–80. doi: 10.1038/s41586-023-06401-0 (PMC10447239; doi:10.1038/s41586-023-06401-0)
Supplement: Supplementary file 2 — Reporting Summary [file 41586_2023_6401_MOESM2_ESM.pdf]

nature portfolio

Corresponding author(s): Geoffrey L. Smith
Jonas D. Albarnaz

Last updated by author(s): Jun 20, 2023

Reporting Summary

Nature Portfolio wishes to improve the reproducibility of the work that we publish. This form provides structure for consistency and transparency in reporting. For further information on Nature Portfolio policies, see our Editorial Policies and the Editorial Policy Checklist.

Statistics

For all statistical analyses, confirm that the following items are present in the figure legend, table legend, main text, or Methods section.

n/a	Confirmed	
☐	☒	The exact sample size (n) for each experimental group/condition, given as a discrete number and unit of measurement
☐	☒	A statement on whether measurements were taken from distinct samples or whether the same sample was measured repeatedly
☐	☒	The statistical test(s) used AND whether they are one- or two-sided<br>*Only common tests should be described solely by name; describe more complex techniques in the Methods section.*
☐	☒	A description of all covariates tested
☐	☒	A description of any assumptions or corrections, such as tests of normality and adjustment for multiple comparisons
☐	☒	A full description of the statistical parameters including central tendency (e.g. means) or other basic estimates (e.g. regression coefficient) AND variation (e.g. standard deviation) or associated estimates of uncertainty (e.g. confidence intervals)
☐	☒	For null hypothesis testing, the test statistic (e.g. F, t, r) with confidence intervals, effect sizes, degrees of freedom and P value noted<br>*Give P values as exact values whenever suitable.*
☒	☐	For Bayesian analysis, information on the choice of priors and Markov chain Monte Carlo settings
☒	☐	For hierarchical and complex designs, identification of the appropriate level for tests and full reporting of outcomes
☒	☐	Estimates of effect sizes (e.g. Cohen's d, Pearson's r), indicating how they were calculated

Our web collection on statistics for biologists contains articles on many of the points above.

Software and code

Policy information about availability of computer code

Data collection	Image Studio Acquisition Software (version 5.2)<br>ZEN Microscope Software (version 6.0.0.485)<br>AxioVision (version 4.8)<br>FLUOstar Omega Reader Control Software (version 1.20)<br>MaxQuant (version 2.0.1.0)<br>QuantStudioTM Real-Time software (v1.3)
Data analysis	GraphPad Prism (version 7.04)<br>ZEN Lite Microscope Software (version 2.5.75.0)<br>ImageJ-Fiji (version 1.53)<br>Perseus (version 1.6.2.1)<br>MARS Data Analysis Software (version 2.00)<br>Image Studio Lite Quantification Software (version 5.5.4)<br>Clustal Omega (1.20)

For manuscripts utilizing custom algorithms or software that are central to the research but not yet described in published literature, software must be made available to editors and reviewers. We strongly encourage code deposition in a community repository (e.g. GitHub). See the Nature Portfolio guidelines for submitting code & software for further information.

Data

Policy information about availability of data

All manuscripts must include a data availability statement. This statement should provide the following information, where applicable:

- Accession codes, unique identifiers, or web links for publicly available datasets
- A description of any restrictions on data availability
- For clinical datasets or third party data, please ensure that the statement adheres to our policy

All data from this study including supplementary material are available.

Proteomic data generated from label-free MS are uploaded in iProX repository, with the dataset identifier IPX0005650001.

Orthopoxvirus nucleotide sequences cited in this study are available on NCBI GenBank: VACV (YP_232972.1 and YP_232904.1), RPXV (AAS49792.1 and AAS49727.1), CPXV (ADZ24099.1 and NP_619819.1), CMLV (NP_570478.1 and NP_570410.1), MPXV UK 2022 (UWM73237.1 and UWM73173.1), MPXV Zaire (NP_536509.1 and NP_536509.1) and VARV major strain India 1967 (APR62813.1 and P0DSX3.1).

Human research participants

Policy information about studies involving human research participants and Sex and Gender in Research.

Reporting on sex and gender	N/A
Population characteristics	N/A
Recruitment	N/A
Ethics oversight	N/A

Note that full information on the approval of the study protocol must also be provided in the manuscript.

Field-specific reporting

Please select the one below that is the best fit for your research. If you are not sure, read the appropriate sections before making your selection.

☒ Life sciences ☐ Behavioural & social sciences ☐ Ecological, evolutionary & environmental sciences

For a reference copy of the document with all sections, see nature.com/documents/nr-reporting-summary-flat.pdf

Life sciences study design

All studies must disclose on these points even when the disclosure is negative.

Sample size

Base on our previous extensive experience studying orthopoxviruses, we selected a sufficiently large sample size for each study as follows:

Virus replication assays were carried out with n = 3 per condition
Virus spread assays were carried out with n ≥ 3 per condition
Virus plaque size measurements were recorded with n ≥ 6
Reporter gene assays were performed with n = 3 per condition, and 3 separate experiments
Immunoblot quantification were from 3 independent experiments
Immunofluorescence quantification was from n ≥ 36 per condition
MS was performed with n = 2 per condition
RT-qPCR was performed with n = 3 per condition, and 3 separate experiments

Data exclusions

No data exclusions in this study.

Replication

Virus replication and spread assays were performed twice, and virus plaque size measurement assays were performed at least three times. Graphs shown are consistent between independent experiments. Reporter gene assays are representative of three independent experiments. Co-precipitation and immunoblotting assays were performed three times and wheat germ transcription/translation assays were performed twice. Blots shown are representative of all repeats. Reporter gene assays are representative of three independent experiments. Immunofluorescence experiments were performed three times. Images shown are representative of all independent experiments. Mass spectrometry was performed once.
All repeat experiments gave the same result.

Randomization

For plaque size quantification, the plaques were picked randomly at each condition.

Randomization is not applicable in the remaining experiments. Cell lines were allocated by genotype and were cultured side by side to minimize unpredicted environmental variations.

Blinding

Blinding was not applicable because this study does not include clinical trials. Researchers were not blinded to different cell lines. Virus infection experiments were conducted independently by Y. Zhao, Y. Lu and S. Richardson, whenever possible.

Reporting for specific materials, systems and methods

We require information from authors about some types of materials, experimental systems and methods used in many studies. Here, indicate whether each material, system or method listed is relevant to your study. If you are not sure if a list item applies to your research, read the appropriate section before selecting a response.

Materials & experimental systems

n/a	Involved in the study
☐	☒ Antibodies
☐	☒ Eukaryotic cell lines
☒	☐ Palaeontology and archaeology
☒	☐ Animals and other organisms
☒	☐ Clinical data
☒	☐ Dual use research of concern

Methods

n/a	Involved in the study
☒	☐ ChIP-seq
☒	☐ Flow cytometry
☒	☐ MRI-based neuroimaging

Antibodies

Antibodies used

Mouse anti-FLAG Sigma-Aldrich Cat# F3165; RRID:AB_259529, dilution 1:1000
Rabbit anti-FLAG Sigma-Aldrich Cat# F7425; RRID:AB_439687, dilution 1:1000
Mouse anti-HA BioLegend Cat# 901513; RRID:AB_2565335, dilution 1:1000
Rabbit anti-HA Sigma-Aldrich Cat# H6908; RRID:AB_260070, dilution 1:1000
Mouse anti-Myc Merck Millipore Cat# 05-724; RRID:AB_11211891, dilution 1:1000
Mouse anti-GAPDH Sigma-Aldrich Cat# G8795; RRID:AB_1078991, dilution 1:1000
Rabbit anti-α-actin Sigma-Aldrich Cat# A2066; RRID:AB_476693, dilution 1:1000
Mouse anti-TRIM5 Santa Cruz Biotechnology Cat# sc-373864; RRID:AB_10918111, dilution 1:1000
Mouse anti-HDAC5 Santa Cruz Biotechnology Cat# sc-133225; RRID:AB_2116791, dilution 1:1000
Mouse anti-IKKβ Merck Millipore Cat# 05-535, RRID:AB_2122161, dilution 1:1000
Rabbit anti-CypA Invitrogen Cat# PA1-025; RRID:AB_2169124, dilution 1:1000
Rabbit anti-p-TAK1 Cell Signaling, Cat# 4508S; RRID:AB_561317, dilution 1:500
Rabbit anti-IkBa Cell Signaling, Cat# 9242S; RRID:AB_331623, dilution 1:1000
IRDye 680RD-conjugated goat anti-rabbit IgG LI-COR Cat# 926-68071; RRID:AB_10956166, dilution 1:1000
IRDye 800CW-conjugated goat anti-mouse IgG LI-COR Cat# 926-32210; RRID:AB_621842, dilution 1:1000
Donkey anti-mouse IgG (H+L) secondary antibody, Alexa Fluor 546 Invitrogen Cat# A10036; RRID:AB_2534012, dilution 1:5000
Goat anti-rabbit IgG (H+L) secondary antibody, Alexa Fluor 488 Invitrogen Cat# A11008; RRID:AB_143165, dilution 1:5000
Donkey anti-rabbit IgG (H+L) secondary antibody, Alexa Fluor 546 Invitrogen Cat# A11010; RRID:AB_2534077, dilution 1:5000
Rabbit anti-C6 Unterholzner et al., 2011 N/A, dilution 1:1000
Mouse anti-D8 Parkinson & Smith, 1994 N/A, dilution 1:1000
Rabbit anti-L3 Resch & Moss, 2005 N/A, dilution 1:1000
Rabbit anti-C6 and Mouse anti-D8 were produced in this lab.
Rabbit anti-L3 was a kind gift from Dr Bernard Moss, NIAID.

Validation

The Rabbit anti-C6 antibody was validated in Unterholzner et al., 2011, the Rabbit anti-L3 in Resch & Moss, 2005, the Mouse anti-D8 in Parkinson & Smith, 1994, and the Mouse anti-HDAC5 in Soday et al., 2019, (Fig. 7D, 7H). The Mouse anti-TRIM5 (Fig. ED 2a, 2b) and the Rabbit anti-CypA (Fig. ED 3a, 3b) antibodies were validated in this study using knockout cell lines lacking TRIM5 or CypA.

Validation detail for the other commercial antibodies is available on the manufacturer's website that is attached as following.
Mouse anti-FLAG Sigma-Aldrich Cat# F3165; https://www.sigmaaldrich.com/GB/en/product/sigma/f3165
Rabbit anti-FLAG Sigma-Aldrich Cat# F7425; https://www.sigmaaldrich.com/GB/en/product/sigma/f7425
Mouse anti-HA BioLegend Cat# 901513; https://www.biolegend.com/en-us/products/anti-ha-11-epitope-tag-antibody-11071?GroupID=GROUP26
Rabbit anti-HA Sigma-Aldrich Cat# H6908; https://www.sigmaaldrich.com/GB/en/product/sigma/h6908
Mouse anti-Myc Merck Millipore Cat# 05-724; https://www.merckmillipore.com/GB/en/product/Anti-Myc-Tag-Antibody-clone-4A6,MM_NF-05-724
Mouse anti-GAPDH Sigma-Aldrich Cat# G8795; https://www.sigmaaldrich.com/GB/en/product/sigma/g8795
Rabbit anti-α-actin Sigma-Aldrich Cat# A2066; https://www.sigmaaldrich.com/GB/en/product/sigma/a2066
Rabbit anti-p-TAK1 Cell Signaling, Cat# 4508S; https://www.cellsignal.com/products/primary-antibodies/phospho-tak1-thr184-187-90c7-rabbit-mab/4508
Rabbit anti-IkBa Cell Signaling, Cat# 9242S; https://www.cellsignal.com/products/primary-antibodies/ikba-antibody/9242
IRDye 680RD-conjugated goat anti-rabbit IgG LI-COR Cat# 926-68071; https://www.licor.com/bio/reagents/irdye-680rd-goat-anti-rabbit-igg-secondary-antibody
IRDye 800CW-conjugated goat anti-mouse IgG LI-COR Cat# 926-32210; https://www.licor.com/bio/reagents/irdye-800cw-goat-anti-mouse-igg-secondary-antibody
Donkey anti-mouse IgG (H+L) secondary antibody, Alexa Fluor 546 Invitrogen Cat# A10036; https://www.thermofisher.com/

antibody/product/Donkey-anti-Mouse-IgG-H-L-Highly-Cross-Adsorbed-Secondary-Antibody-Polyclonal/A10036
Goat anti-rabbit IgG (H+L) secondary antibody, Alexa Fluor 488 Invitrogen Cat# A11008; https://www.thermofisher.com/antibody/product/Goat-anti-Rabbit-IgG-H-L-Cross-Adsorbed-Secondary-Antibody-Polyclonal/A-11008
Goat anti-rabbit IgG (H+L) secondary antibody, Alexa Fluor 546 Invitrogen Cat# A11010; https://www.thermofisher.com/antibody/product/Goat-anti-Rabbit-IgG-H-L-Cross-Adsorbed-Secondary-Antibody-Polyclonal/A-11010

Eukaryotic cell lines

Policy information about cell lines and Sex and Gender in Research

Cell line source(s)	Human foetal foreskin fibroblasts (HFFFs) immortalized with human telomerase (HFFF-TERTs) was a kind gift from Prof Michael Weekes (University of Cambridge), who obtained them from Prof Richard Stanton (Cardiff University), PMID: 17522202.<br>BS-C-1 (African green monkey cell line) ATCC, ATCC: CCL-26<br>RK13 cells (rabbit kidney cell line) ATCC, ATCC: CCL-37<br>HeLa (human cervical adenocarcinoma epithelial cell line) ATCC, ATCC: CCL-2<br>HEK293T (human embryo kidney epithelial cell line) ATCC, ATCC: CRL-11268<br>T-REx-293 Life technologies, R71007<br>CRISPR Ctrl HeLa (for TRIM5-/-) This paper<br>TRIM5-/- clone 1 HeLa This paper<br>TRIM5-/- clone 2 HeLa This paper<br>CRISPR RICE T-REx-293 (for TRIM5-/-) This paper<br>TRIM5-/- clone 1 T-REx-293 This paper<br>TRIM5-/- clone 2 T-REx-293 This paper<br>EV T-REx-293 in T5-/- (complementation) This paper<br>TAP-T5α WT T-REx-293 (complementation) This paper<br>TAP-T5γ WT T-REx-293 (complementation) This paper<br>TAP-T5δ WT T-REx-293 (complementation) This paper<br>EV T-REx-293 (over-expression) This paper<br>FLAG-T5α WT T-REx-293 (over-expression) This paper<br>FLAG-T5γ WT T-REx-293 (over-expression) This paper<br>FLAG-T5δ WT T-REx-293 (over-expression) This paper<br>FLAG-T5α L19R T-REx-293 (complementation) This paper<br>FLAG-T5α N70A T-REx-293 (complementation) This paper<br>FLAG-T5α R119E T-REx-293 (complementation) This paper<br>FLAG-T5α ΔSPRY T-REx-293 (complementation) This paper<br>CRISPR/Cas9 control T-REx-293 (for CypA-/-) This paper<br>CypA-/- clone 1 T-REx-293 This paper<br>CypA-/- clone 2 T-REx-293 This paper<br>EV T-REx-293 in CypA-/- (complementation) This paper<br>TAP-CypA WT T-REx-293 (complementation) This paper<br>TAP-CypA R55A T-REx-293 (complementation) This paper<br>TAP-CypA F113A T-REx-293 (complementation) This paper<br>CRISPR RICE T-REx-293 (for TRIM5-/- CypA-/-) This paper<br>T5-/- CypA-/- clone 1 T-REx-293 This paper<br>T5-/- CypA-/- clone 2 T-REx-293 This paper<br>EV T5-/- CypA-/- T-REx-293 (complementation) This paper<br>TAP-CypA T5-/- CypA-/- T-REx-293 (complementation) This paper<br>Myc-VACVC6 HEK293T (over-expression) Soday et al., 2019<br>TAP-VACVB14 HEK293T (over-expression) Lu et al., 2019<br>TAP-VACVcoC6 HEK293T (over-expression) Lu et al., 2019<br>TAP-MPXVUK22B14 T-REx-293 (over-expression) This paper<br>TAP-VACVcoC6 T-REx-293 (over-expression) This paper<br>TAP-RPXVC6 T-REx-293 (over-expression) This paper<br>TAP-CPXVC6 T-REx-293 (over-expression) This paper<br>TAP-ElephantpoxvirusC6 T-REx-293 (over-expression) This paper<br>TAP-CMLVC6 T-REx-293 (over-expression) This paper<br>TAP-MPXVUK2022coC6 T-REx-293 (over-expression) This paper<br>TAP-MPXVZairecoC6 T-REx-293 (over-expression) This paper<br>TAP-VARVcoC6 T-REx-293 (over-expression) This paper<br>TAP-TAPcoL3 T-REx-293 (over-expression) This paper
Authentication	Commercial cell lines were authenticated by the suppliers, detail of the authentication is provided as following,<br>BS-C-1 (African green monkey cell line) ATCC: CCL-26, https://www.atcc.org/products/ccl-26<br>RK13 cells (rabbit kidney cell line) ATCC: CCL-37, https://www.atcc.org/products/ccl-37<br>HeLa (human cervical adenocarcinoma epithelial cell line) ATCC: CCL-2, https://www.atcc.org/products/ccl-2<br>HEK293T (human embryo kidney epithelial cell line) ATCC: CRL-11268, https://www.atcc.org/products/crl-11268<br>The above cell lines were authenticated by ATCC, and a link to each cell line describing the authentication is attached.<br>T-REx-293 Life technologies, R71007 were authenticated by Life technologies. https://www.thermofisher.com/order/catalog/product/R71007<br><br>HFFFs were tested regularly to confirm that human leukocyte antigen (HLA) and MHC Class I Polypeptide-Related Sequence A (MICA) genotypes, cell morphology and antibiotic resistance are unchanged. In addition, HCMV only replicates in human fibroblast cells (dermal or foreskin in origin) and so HCMV infection was tested and confirmed in HFFF cells.

Mycoplasma contamination	The authors confirm that all cell lines tested negative for mycoplasma contamination.
Commonly misidentified lines (See ICLAC register)	No commonly misidentified cell lines were used in this study.
